# Child health nurse perceptions of using confident body, confident child in community health: a qualitative descriptive study

**DOI:** 10.1186/s12912-020-00499-7

**Published:** 2020-11-06

**Authors:** Lyza N. Norton, Laura M. Hart, Francoise E. Butel, Shelley Roberts

**Affiliations:** 1 Gold Coast Hospital and Health Service, 1 Hospital Blvd, Southport, QLD 4215 Australia; 2 School of Psychology and Public Health, La Trobe University, Bundoora Campus, Bundoora, VIC 3086 Australia; 3 Centre for Mental Health, Melbourne School of Population and Global Health, University of Melbourne, Melbourne, VIC 3010 Australia; 4 School of Allied Health Sciences, Griffith University, Gold Coast Campus, Southport, QLD 4222 Australia

**Keywords:** Body image, Child health nurses, Children, Eating disorder prevention, Nutrition, Primary care, Public health

## Abstract

**Background:**

Confident Body, Confident Child (CBCC) is an innovative, evidence-based program providing parenting strategies to promote healthy eating, physical activity and body satisfaction in children aged 2–6 years. This study aimed to explore Child Health Nurse (CHN) experiences with using CBCC in their community health clinics with parents of young children. This work is part of a larger study involving tailoring, implementing and evaluating CBCC in a community child health setting.

**Methods:**

This qualitative descriptive study was conducted within community child health centres at a public health service in Queensland, Australia. Participants included CHNs who had recently attended a tailored CBCC training workshop providing training/education, group activities/discussions and CBCC resources for CHN use in clinical practice. Semi-structured interviews were conducted to explore CHN perceptions of CBCC training, content and resources; and how CBCC was used in practice. Interviews were recorded and transcribed verbatim and analysed thematically.

**Results:**

Eleven CHNs participated in interviews, with three themes emerging from the data. In Theme 1, *High CHN satisfaction with CBCC messages, resources and utility*, nurses expressed CBCC was highly valuable, useful and easy to enact in their practice. In Theme 2, *Effects of CBCC on CHN knowledge, behaviour and practice*, CHNs said they experienced increased awareness around body image, improved confidence in addressing issues with clients, and positive changes in their own behaviour and practice after attending CBCC training. In Theme 3, CHNs discussed *Ideas for future implementation of CBCC*, including challenges and considerations for practice, ongoing education/training for CHNs and broadening the target audience for wider CBCC dissemination.

**Conclusions:**

This study found CHNs were highly accepting of CBCC as it was useful and valuable in practice, increased their awareness and confidence around body image issues, and positively affected their attitudes and behaviours. CHNs’ suggestions for making CBCC delivery more efficient and broadening its reach in the community were valuable and will likely inform local policy and future research. Further research is required on the wider dissemination of CBCC to parents of young children for promoting positive body image and healthy eating, ultimately for the long-term prevention of eating disorders.

**Supplementary Information:**

The online version contains supplementary material available at 10.1186/s12912-020-00499-7.

## Background

Eating disorders affect 5–9% of Australians [1, 2] and place significant burden on patients, families and health care systems [3, 4]. Poor body image is a predictor for the development of disordered eating [5, 6], the foundations for which develop in early childhood [7]. Parents have the most significant impact on their child’s learned attitudes and behaviours around body image and eating patterns [8], hence prevention programs should target parents of young children. Confident Body, Confident Child (CBCC) is a program that educates parents and equips them with tools and strategies to promote positive body image and eating patterns with their children [9]. A RCT showed CBCC was effective in improving parents’ knowledge, intentions and behaviours; and in increasing body satisfaction among children [9]. However, its feasibility and impact in a community health setting is yet to be determined.

To achieve universal eating disorder prevention, CBCC needs to be delivered at a population level. One way to achieve this is to embed CBCC within existing health services. In Australia, primary paediatric care includes key developmental surveillance health checks conducted by community Child Health Nurses (CHNs). CHN community clinics are funded by State and Local Government Areas for children from birth to school age. Each child has access to at least five CHN consultations in their first year of age, followed by another three before school age (at 18 months, 2.5–3.5 years and 4–5 years); with around three-quarters of families in the health service’s catchment area accessing the services [10]. Due to their broad reach in communities, CHNs are in an optimal position to deliver CBCC messages and resources to parents during their existing clinic appointments. Up-skilling CHNs to disseminate CBCC is likely to be an effective, efficient and sustainable way to invest in the promotion of healthy body image and eating patterns, and ultimately, in the prevention of eating disorders, among young children. With increased paediatric admissions for eating disorders at our health service, CBCC was identified as an ideal program for broad-reaching, upstream prevention.

Our team has adapted CBCC to the needs of community CHNs at a health service in Australia, to enable delivery of CBCC messages and resources to parents of children aged 2–6 years in their existing clinics, in which 30,000 occasions of service are provided each year [10]. To achieve rapid translation into practice, an effectiveness-implementation hybrid design [11] was used to simultaneously evaluate implementation (process evaluation; the focus of the current paper) and effectiveness (outcome evaluation) of CBCC. Medical Research Council guidance on developing and evaluating complex interventions recommends conducting thorough process evaluation alongside implementation of health interventions, to gain insight into whether interventions work or not, and why [12]. This study reports on an aspect of process evaluation; participant responses to and interactions with the intervention.

The aim of this study was to explore CHN experiences with using the CBCC program in their community health clinics. The perspectives of CHNs are critically important in understanding the feasibility and utility of CBCC being embedded within community health care services.

## Methods

### Study overview

This qualitative descriptive study was part of a larger project evaluating CBCC implementation in an Australian health service district. Implementation of an adapted CBCC program involved providing CBCC training and resources to CHNs in a 1-day workshop; and encouraging CHNs to use CBCC in their clinics with parents of 2–6-year-old-children. Semi-structured interviews were conducted with CHNs following implementation, to explore their experiences with using CBCC in their usual practice. The study received ethical approval through relevant hospital and university human research ethics committees (reference numbers masked for blinded peer review).

### Intervention

CBCC is an evidenced-based, educational program originally designed for parents of 2–6-year-old children, providing them with strategies for promoting positive body image, healthy eating and physical activity in their children [9]. Our multidisciplinary team of academic and clinician researchers worked with stakeholders to adapt the existing CBCC program for CHNs to deliver CBCC messages and resources to parents during their usual clinics. The adapted intervention involved a full-day training workshop for CHNs, co-led by two members of the research team who were trained CBCC facilitators (LN, a senior Paediatric Dietitian within the health service; and LH, a research fellow in mental health who co-developed CBCC). The workshop covered the three key CBCC topics: 1) child body image, with strategies on how to develop healthy body attitudes (e.g. by avoiding weight stigma and appearance-based comments); 2) healthful eating attitudes and sustainable eating patterns, with strategies for encouraging intuitive eating in children, family meals, and managing ‘sometimes’ foods and fussy eating; 3) healthy body weight and strategies for avoiding diet culture, managing screen time and encouraging regular physical activity. The workshop was presented via a structured PowerPoint presentation, small group discussions and group activities. CHNs were provided with CBCC parent resource packs for use in clinics, which included: a graphically designed A5-sized ‘Parent Book’; ‘Extended Family Book’; ‘Do/Don’t’ Poster; children’s picture book ‘Shapesville’ (effective in improving child body image [13]); and leaflet with website link (the website provided the Parent Book in electronic form, factsheets, further reading, videos, and activities). CHNs were given access to factsheets that could be printed out from the CBCC website and given to parents to take home. In addition, a quick reference guide poster was designed for CHNs to help them navigate CBCC resources and have open dialogue with parents on the key CBCC topics (body image, healthful eating patterns and healthy weight).

### Study setting and participants

The study was conducted within a single health district including 11 separate Community Child Health (CCH) centres in Queensland, Australia. The district employs 46 CHNs (across 35 full time equivalent positions) in CCH centres, providing services such as health and developmental checks; hearing assessments; feeding/nutrition support and information; and education/support groups for children from birth to school age and their parents. CHNs’ professional development includes monthly 1–2-h training sessions; and three 1-day training workshops annually, on topics decided by CHN managers. CHNs employed in CCH centres across the district were invited to attend a full-day CBCC training workshop in May 2019 (as part of the larger study) by emailing them a flyer. CHN managers also promoted workshop attendance at staff meetings. Demographic data were collected from the 26 CHNs who attended the workshop, via online surveys using Research Electronic Data Capture (REDCap). Around five months after the workshop was delivered, two members of the research team (LN and SR) attended an unrelated, full-day CHN team meeting. At this meeting, nurses who had attended the previous CBCC training workshop were invited to an interview, to discuss their perceptions of the program. Eleven CHNs were recruited using maximum variation purposive sampling. All were female with mean age of 51.4 (±10.5) years (range 35–68 years). Ten of the 11 CHNs reported having two or more children and one had two grandchildren. Eight participants held a bachelor’s degree and one also had a master’s degree. Participants worked full-time (45.5%) or part-time (54.5%) and had on average 16.8 years of experience in their current role (range 5–40 years’ experience).

### Study procedure

Data were collected by two members of the research team with experience in semi-structured interviews (LN and SR). For CHNs recruited at the team meeting, interviews were conducted one-on-one, face-to-face, in a quiet room and recorded with a handheld digital device. A semi-structured interview guide was developed to elicit a conversational style of interviewing that allowed participants to express their perceptions of, and experiences with using, CBCC (see Supplementary File 1). Questions were asked under four main domains, including: 1) overall experience with CBCC; 2) remembering and understanding CBCC training; 3) using CBCC; and 4) perceived value of CBCC. Interviewers started with a broad question (e.g. ‘what did you think about the CBCC program overall?’) to allow participants to discuss what stood out to them. Through active listening, interviewers used participants’ responses to guide the direction of the conversation and determine which prompts/questions to use next. Data were collected until saturation was achieved (defined as no new ideas or concepts emerging), determined via qualitative analysis of verbatim transcripts.

### Data analysis

Interview audio files were transcribed verbatim and analysed using inductive thematic analysis, following the six-step methodology described by Braun and Clarke [14]. First, the lead author (LN) read and reread transcripts to become familiar with and immersed in the data. Second, key quotes were highlighted and initial codes were generated based on participants’ verbatim statements by LN (e.g. *‘it’s increased my awareness of language’* (CHN 1) coded as ‘increased awareness of language’). Third, LN used a qualitative analysis computer software package (NVivo, QSR International, v.11) to group codes, according to similarity, into potential sub-themes; ensuring all data were captured. Fourth, refinement of these potential sub-themes, was performed at two levels. At Level 1, another author (SR) reviewed all collated codes under each potential sub-theme to consider whether they formed a coherent pattern, resulting in some amendments to initial sub-themes (e.g. some sub-themes were merged into one when overlap was identified; whilst others were split when there was too much variation in grouped codes). At Level 2, LN and SR together reviewed individual sub-themes in relation to the entire data set, to generate a thematic ‘map’ of the analysis, which resulted in the sub-themes being grouped according to similarity under broader concepts (these groupings became themes). Throughout step four, LN and SR constantly referred back to the entire data set, 1) to ascertain whether the themes and sub-themes accurately represented the dataset; and 2) to ensure no data were missed in earlier coding stages. In step five, LN and SR continued to revise and refine the specifics of each theme and sub-theme in an iterative way, until all data were adequately represented, and the overall ‘story’ of the data was clear. Clear definitions and names for each theme and sub-theme were generated and continued to be revised iteratively with LH’s input. Finally, step six involved selecting valid and compelling participant quotes to support each sub-theme, and presenting the data in a logical way, which LN, SR and LH were all involved in.

## Results

Participant responses formed three themes, each with multiple sub-themes, as outlined in Table 1. Each theme and sub-theme, with supporting verbatim quotes, are described in detail below.

**Table 1:** Results of inductive qualitative data analysis

Themes	Sub-themes
1. High CHN satisfaction with CBCC messages, resources and utility	a. Valuing CBCC and its key messages
	b. Usefulness of CBCC resources
	c. Enacting CBCC in practice is easy and worthwhile
2. Effects of CBCC on CHN knowledge, behaviour and practice	a. Heightened awareness of body image and language
	b. Positive changes to nurses’ own attitudes, behaviours and clinical practice
	c. Increased confidence in identifying and addressing parental body image issues
3. Ideas for future implementation of CBCC	a. Considerations and challenges for using CBCC in practice
	b. Continuing education and training for CHNs
	c. Broadening target audience for wider dissemination of CBCC

### High CHN satisfaction with CBCC messages, resources and utility

The first theme portrays CHNs’ high satisfaction with CBCC, including its key messages, its resources, and its utility in practice when used with parents in clinics; as described in detail in the three sub-themes below.

#### Valuing CBCC and its key messages

Overall, CHNs were overwhelmingly positive about CBCC. They thought it was *“an excellent program”*, *“loved the concept”* and found it highly interesting, important and valuable. Many nurses expressed satisfaction that these topics were being addressed as they felt it was *“not out there”* currently:

*I think it’s absolutely awesome that we’re actually starting to talk about it… the ripple effect is huge, and I think we just need to be talking about it in as much of our contact [with] parents as possible.* (CHN10)

CHNs valued the fact that CBCC was evidence-based, and suggested ongoing research in this area was important. Nurses said CBCC was relevant, useful and valuable to their practice; and many spoke about embedding it in their clinics. Nurses seemed to take ownership of CBCC; as one explained, she *“really grabbed on to it”* and started using it in practice, while another said the topics aligned with CHNs’ core work and CBCC made current practice more efficient:

*I think a lot of the [CBCC concepts], child health nurses have been doing quite a lot of it anyway, and [CBCC] has basically…streamlined it and given some extra resources that are more specific to that age group.* (CHN5)

CBCC’s key messages and strategies seemed to resonate with CHNs. They often mentioned how CBCC encouraged importance of family meal-times and the language used around body image. They thought CBCC was helpful to parents as it equipped them with the knowledge and confidence they needed to identify and address body image issues with their children. CHNs said most parents were interested in CBCC and had given positive feedback on the program.

*I really liked the concept that this is something that starts at an early age and I talked to parents about changing the world, one family at a time.* (CHN 11)

#### Usefulness of CBCC resources

CHNs described how useful each of the CBCC resources were and how they intended to keep using them after the project finished. Several nurses thought all parents should have access to the resources, or at the very least, the parent book. Nurses’ perceptions on each CBCC resource are outlined below.

The *‘Dos and Don’ts’ poster* was described as very useful. CHNs found it a convenient, easily accessible, “*short and snappy”* reminder for themselves; and a good overview for parents of CBCC messages. CHNs liked having the poster on clinic walls so parents could read it when waiting, or so nurses could show parents on the way in/out of appointments, which nurses said assisted them in delivering CBCC messages. CHNs said parents liked the poster too. One nurse said she printed posters in a larger size and put them up in several clinics, which other nurses mentioned was very helpful.

*The poster’s huge and we’ve got them blown up everywhere – and not just for [parents] to see, but it’s actually a good reminder for us… when [parents are] in the room, because you can’t remember everything.* (CHN 10)

The *Shapesville book* was another popular resource. Several nurses said they’d used it in their clinics; either by reading it to children or showing it to parents/grandparents. Nurses thought it was *“fantastic”* for educating children on body acceptance and normalising different shapes and sizes. Many nurses wanted more copies of Shapesville for their clinics/waiting rooms, and some mentioned their team leader had ordered it for local council libraries.

*That little Shapesville book is fantastic, because often the parents will sit there and have a flick through that while I'm doing the hearing test.* (CHN5)

CHNs also liked the *Parent Book* as it contained valuable information and gave parents good ideas on how to discuss sensitive topics with their child/ren. CHNs found the parent book useful for time-poor families, as it was a quick and easy way to deliver CBCC messages to parents. The *CBCC leaflet* was used by several CHNs to refer parents to the CBCC website (where they could access more information or order CBCC resources if they wished). The *website* was used widely by nurses, who described it as easy to access with lots of information. It was especially useful if parents showed interest in CBCC and wanted more information. All three resources mentioned here were especially useful to nurses when time was a barrier in appointments. One nurse said she found *open dialogue* most beneficial, as she was worried that parents (especially those with body image issues) may feel personally attacked if given too many resources.

#### Enacting CBCC in practice is easy and worthwhile

CHNs seemed to take ownership of CBCC content, expressing that it aligned with the topics covered in their usual care and helped to improve care delivery. One nurse said CBCC *“should be our bread and butter…it needs to be in our toolkit, and everyone should be comfortable with it… it needs to be one of those things that we take with us wherever we go”* (CHN 10).

Most nurses thought CBCC concepts were easy to bring into conversations with parents in their usual clinics, and in some cases were a priority, *“because this is what’s come up, and this is actually the most important thing, not [measuring] weights and lengths”*. Many CHNs thought CBCC topics could be incorporated into parent education groups. Nurses were highly accepting of CBCC and were happy to try something new, because it met their needs as clinicians. They used CBCC to reassure, promote and educate parents. For example, they reassured parents that their child’s weight was within normal limits; promoted body acceptance and healthy eating; and educated parents on using positive language around food, weight and body image. The desire to embed CBCC in practice was evident, with nurses describing CBCC becoming *“part of our language”*. Many nurses said they wanted to continue using CBCC resources after the research project finished, and others expressed an urgency to do more. Several nurses felt they didn’t have the opportunity to use CBCC as much as they would have liked to, due to the area in which they were currently working, such as with infants instead of pre-schoolers.

### Effects of CBCC on CHN knowledge, behaviour and practice

CHNs discussed the effects they perceived CBCC training to have had on their own attitudes, behaviours and clinical practice, as well as the effects of implementation they saw on parents. In sub-theme a), CHNs described increased awareness of body image issues and in particular, language used around eating, weight and appearance, after attending training. This awareness led to improved attitudes and behaviours towards body image, eating and weight (in sub-theme b). Nurses then felt they could role model positive ways to speak about body image and healthy eating to parents, who could then do the same with their children (sub-theme c).

#### Heightened awareness of body image and language

CHNs strongly expressed that CBCC greatly improved their awareness around body image issues, including the link between dieting behaviours and increased risk of eating disorders. One was shocked to learn how young some children were diagnosed with eating disorders, describing it as *“scary”*. Most nurses said the training workshop raised new content with them, as the topic was *“not something I suppose I’d given much thought to before”*. Nurses frequently discussed how they became acutely aware of the language they used around eating, body image and weight after attending the training workshop. Many said it led them to self-reflection; once they became aware of their own language, they found themselves thinking about how it could affect others. This awareness was the most notable and frequently mentioned outcome of the workshop by CHNs, who said *“being aware…it’s changed my conversation a lot of the time”* (CHN 9). Many nurses reported reflecting on their language/behaviour, both in their personal lives and at work. As one nurse put it, *“you’ve got to be aware of it before you can change it”.*

*Even in my private life I'm more aware now since doing that course of the things that I say…I didn’t realise how much I was focusing on the physical.* (CHN 4)

#### Positive changes to nurses’ own attitudes, behaviours and clinical practice

Many CHNs reported that they had made significant changes to their own behaviour at home in their personal lives, and in clinical practice, as a result of CBCC training/implementation. Some explained how they incorporated CBCC concepts into everyday life at home with friends and family: *“personally, just not running myself down in front of my kids as much”*. Others said they’d made changes to general conversations, and were trying to avoid complimenting others on their physical appearance. A few nurses explained how useful CBCC could be for their own parenting:

*I'm just rapt to have this, because I've got two daughters myself. So, it's a real eye-opener for me and how I'm using my language at home. It's been really beneficial for me personally, as well as professionally.* (CHN 8)

Many CHNs described how they had changed the way they spoke to parents/children in their clinics, being more aware and mindful of their language, as a result of CBCC training. Some said they used to regularly comment on clients’ appearance (e.g. ‘you look great’ to postpartum mothers) or give children appearance-based compliments; but were now conscious of how such comments might reinforce the idea that appearance is of primary importance. CHNs described how they now tried to avoid such comments to ensure they had positive impacts on their clients. Other ways in which nurses changed their language included using the word ‘growth’ rather than weight gain’ and ‘nutritional needs’ instead of ‘diet’ when talking to parents about their child. Nurses suggested *“parents are quite unaware of the subliminal messages that they might be passing on to their kids”* just with their language. Nurses made an effort to highlight this to parents (and colleagues), for example by *“trying to teach them, and role modelling the correct ways to speak to children”* (CHN 4). Nurses said that some parents had realisations about their language once they were educated about it:

*“I guess, just making parents aware of their words and actions that can impact their children's behaviour, that potentially they hadn't given any thought to”* (CHN 11).

#### Increased confidence in identifying and addressing parental body image issues

Nurses explained how the knowledge and skills they had acquired from CBCC training enabled them to identify issues they may have previously overlooked and increased their confidence in addressing such issues; specifically, in the context of parental body image. Several nurses said they could now ‘read between the lines’ and identify ‘red flags’ among mothers who may have eating or body image issues, by the way in which mothers discussed their own eating patterns and weight. CHNs were mindful of how these mothers’ body attitudes could affect their child. Some nurses said they now asked mothers about their own eating behaviours when relevant; and targeted education at mothers (in order to benefit the child), by explaining that children learn eating patterns and body image from their parents.

*I've had a conversation with a mum…because she'd used food as treats…it's like well, how about treat, can we go to the park instead of around food. She was really receptive because she has got quite a lot of issues, body image issues herself.* (CHN 8)

Nurses explained that new mothers were particularly vulnerable to poor body image, having recently given birth and feeling pressure to get back to their pre-baby weight. This was seen by nurses as a critical time to educate mothers, not just for the mothers’ own sake, but for the sake of their children too, especially if the new baby had older siblings. When nurses perceived body image to be a sensitive topic for mothers, they sometimes opened dialogue by first discussing the child’s behaviour of concern:

*Sometimes…you can open dialogue through the child. Because sometimes the child is mimicking mum with body image… I was able to come in that way and then [say], “how healthy is that for that child at this age to be doing that?” They then go back, they've either had some trauma, or they were forced to eat when they were kids, or something comes up from it, and then you can explore it better.* CHN 7

CHNs reported increased confidence in discussing the key CBCC messages with parents after attending the training workshop. Nurses felt empowered by the knowledge and awareness they’ had gained, which allowed them to confidently and comfortably discuss sensitive issues with parents, especially body image:

*I feel comfortable talking about the language used around body image; that’s a really good start for young kids… and [I have] increased confidence in educating parents on language used with children.* (CHN 3)

Nurses explained how in some cases, their confidence in addressing arising issues with parents depended on the rapport they had with them:

*One of my mums brought up the fact that she started a keto diet and there is no bread in the house for this 10-month old. I was like “okay, we need to have a chat about this”, which she was really receptive [to] and she took it really well. Because we have that relationship, I can challenge that a little bit.* (CHN 8)

However, CHNs said that CBCC content was difficult to broach with some parents. They desired more training on how to start these tricky conversations (described in more detail in Theme 3). Also, despite feeling more confident in discussing body image and delivering CBCC content to parents after the training, some CHNs still wanted confirmation from other nurses or health care professionals to reassure them that they were doing the right thing.

*Sometimes I feel like you just need someone there in your area if you’re having trouble, to say, “look this is what I’ve done, what’s the next step?”* (CHN 3)

### Ideas for future implementation of CBCC

In the final theme, nurses discussed ideas for implementing CBCC more broadly, effectively and efficiently in the future as part of regular service delivery. This included: a) considerations and challenges for using CBCC in usual practice; b) the need for ongoing training and support; and c) widening the target audience and setting for disseminating CBCC.

#### Considerations and challenges for using CBCC in practice

CHNs acknowledged some topics covered by CBCC were potentially sensitive or controversial (especially body image and dieting behaviours). Nurses considered these topics particularly difficult to discuss with certain subgroups of parents, e.g. parents with high body weight; parents they hadn’t met before/lacked rapport with; parents with strong beliefs about diets (e.g. vegan or ‘keto’ diets); or parents who had their own body image issues. Nurses reported feeling uncomfortable, awkward or just not knowing how to broach the subject in these cases.

*Sometimes…parents are hard to open that dialogue [with]…the ones who are closed and they've got their own body image issues, then that's difficult, I find.* (CHN 7)

However, other CHNs suggested ways to overcome these challenges, such as building rapport with parents before broaching sensitive issues, as having a good relationship allowed nurses to speak more openly and assess parents’ readiness for such discussions. Several CHNs thought timing was key: *“It’s always a controversial topic…timing is really important, (and) being aware that if a parent is overweight…it’s going to be trickier to talk about”* (CHN 3). Some nurses said they would focus more on food and eating if speaking about body image was uncomfortable for parents. Other CHNs spoke about strategies such as ‘layering’ information, so as not to overwhelm parents:

*You start with little bits, but you’re layering it as the time goes on…you don’t have to overwhelm them. It’s just showing the importance of it and then just gradually, and they’re more likely to ask you questions anyway.* (CHN 10)

Another challenge to CHNs using CBCC in practice was time. Nurses wanted to go into more depth with certain topics, or use CBCC with all parents and children, but reported they often didn’t have the time to do so. One CHN highlighted that *“the more you use it, the better you’re going to be and the quicker you’re going to be”,* in terms of delivering CBCC in clinics. Nurses also spoke about prioritising topics for each family individually:

*There is so much they want us to do in that small time - but if the priority of that family would be nutrition and absolutely then it would flow.* (CHN 6)

#### Continuing education and training for CHNs

CHNs strongly expressed a desire for ongoing education and training in CBCC. They suggested refresher courses would be helpful to consolidate their knowledge and skills in its delivery to parents and children; and wanted regular updates on the latest research to ensure their practice was evidence-based and up-to-date:

*I think definitely refreshers along the way, and then updates of what's new out there as well. Diets change, so it would be nice to know what the next fad is. That would be helpful.* (CHN 8)

Nurses also wanted more training on how to comfortably start conversations with parents for whom CBCC topics were considered difficult or sensitive; for example body image and eating patterns. *“It would be good to have some more training, because...it’s a delicate topic for [some parents]. You know, it’s probably a delicate topic for us.”* (CHN 7). As another nurse said: *“We need to work out how to talk about it in a friendly, non-judgemental way”* (CHN 9). Some CHNs wanted opportunities to discuss CBCC use in practice with their colleagues, using real life examples of how others incorporated it into conversations, or ‘case studies’ of how other CHNs had successfully tackled difficult scenarios. The idea of peer feedback in a group forum setting was also raised:

*So maybe we should have an open forum where you just come along, you can bring a couple of cases if you want. Because that would be beneficial…you've got a case and… you go, “okay so we've really got a problem here”. Then you can actually share it, how would you have dealt with it?* (CHN 7)

Nurses also suggested ways to streamline CBCC delivery, starting with the identification and prioritisation of families needing CBCC education, through screening tools or flow charts:

*I would really like a tick and flick…[and] maybe a flow chart of where to go…if you’ve ticked it, this is what you do. Maybe that might be helpful.* (CHN 3)

#### Broadening target audience for wider dissemination of CBCC

CHNs in this study provided care to children across a range of ages, from birth to school age. Despite being designed for parents of pre-schoolers from 2 to 6 years, many nurses thought CBCC was relevant to parents of children of all ages, and consequently felt that it should be used more widely among children of different age groups. Some CHNs said CBCC concepts would fit in 6-month old clinics and parent groups, when discussions around introducing solids were frequent:

*I think it would be really good if we could get it in there with us introducing solids. I think parents would really feel that would empower them to then give that positive food and going on right from the outset.* (CHN 2)

Others thought CBCC could be incorporated into existing parent groups, or thought it would be worthwhile to invest in a dedicated parent group or workshop to CBCC:

*I'd love to see it run as a group that parents can go to. Particularly if they've raised issues to say, “yeah, I'm actually struggling with this a bit at home”, you can say, “well, this is a great course you can do”.* (CHN 11)

Many nurses thought school age was an optimal time to instil positive body image in young children as they were impressionable, and starting to learn about the world and themselves. Nurses thought CBCC could be integrated into schools easily and efficiently, considering they were educational institutions with teachers and other staff who could be trained in CBCC: *“I think maybe workshops within schools, like the school-based youth nurses - did you invite them to the training, or have they got a training?”* (CHN 4).

Teenagers were another age group at which CHNs thought CBCC could be targeted. Nurses described teenagers as being impressionable and vulnerable to poor body image, particularly with the influences of social media and peer pressure. While most CHNs preferred CBCC to be introduced at an early age, they saw value in promoting it among teenagers:

*I think even the teens –I know that they've probably already had 12 years of life…but I think at that age they're still capable of change. Just having another positive role model training for them would be a good target opportunity… it's their most impressionable age, where they're hardest on themselves and social media and all of that.* (CHN 4)

Other areas in which CHNs believed CBCC could be disseminated or incorporated into usual business included school and council libraries, school curriculum, kindergartens, formal and informal playgroups, and general practices. Some nurses thought CBCC messages should be *“everywhere”* and part of our *“culture”*, including in the media:

*[CBCC books] should be on the reader list of the schools, for all kids and all parents and it should be…part of the school package…from a very early age. I know there'd be an expense but…the libraries get a lot of money to provide reading services.* (CHN 5)

## Discussion

This study explored CHNs’ experiences with using the CBCC program, including its key messages and resources; the training workshop; implementing it in practice; and ideas for the future. Overall, CHNs were overwhelmingly accepting of and satisfied with CBCC. The key messages resonated with the nurses and they reported finding it extremely valuable both in their personal lives and in their practice. Nurses expressed a desire to continue using CBCC and suggested ideas for broadening and sustaining CBCC use at the population level.

One of the strongest and most prevalent ideas to emerge was a perceived increase in CHNs’ awareness of the language they used around body image, eating and weight. Nine of the eleven nurse participants discussed this, with many saying it was the first step in changing their behaviour and practice. Increasing knowledge and awareness is a fundamental component of behaviour change theoretical models; for example, the Theoretical Domains Framework (TDF) [15]; which is used in behaviour change and implementation research. Knowledge and the beliefs about the consequences of a behaviour are two key influences on human behaviour, according to the TDF. In this study, CHNs frequently spoke about being more aware of their language and how it might impact others’ body image, such as their friends, family and clients (parents and children). The awareness that their language may impact their clients seemed to highly motivate nurses to change their behaviour, both at home and in clinical practice. In previous CBCC research, Hart et al. [16] focused on improving parenting knowledge and in turn, parenting intentions and behaviours, by providing education and strategies for encouraging positive body image with their children. In our study, CHNs said they felt more confident with speaking to parents about CBCC topics after attending the training, as they had acquired new knowledge and skills; and felt better equipped to deliver CBCC messages, having resources on hand. This aligns with another two domains of the TDF affecting behaviour: skills, and beliefs about capabilities [15]. Nurses in our study expressed increased confidence in identifying and addressing body image issues with clients after attending the training, which seemed to increase their use of CBCC in practice. Not all CHNs were confident in all situations, however. Several nurses felt uncomfortable or unsure of how to broach CBCC topics with parents who were had high body weight, had alternative food beliefs or body image issues, or for whom nurses lacked rapport. This is consistent with a previous study, which found health care providers had difficulty discussing a child’s weight if parents were larger-bodied [17]. CHNs in our study expressed a desire for further training specifically focusing on how to raise sensitive topics with parents, case study examples of successful scenarios, and open group discussions with colleagues on ways to approach conversations.

Interestingly, CHNs took ownership of CBCC and immediately incorporated it into their usual practice, despite implementation research suggesting that new innovations or guidelines are difficult to translate into practice, often taking years or decades [18–20]. Nurses in this study explained they were willing to try something new and change their usual practice, as CBCC aligned with their needs, roles and core business as community health professionals, consistent with TDF domain ‘professional role/identity’. Rogers’ theory on the diffusion of preventative innovations also acknowledges this concept, termed ‘compatibility’; the degree to which innovations are perceived to align with users’ values, past experiences, and needs [21]. CHNs frequently discussed how they valued and used CBCC messages and concepts both in their personal lives and in clinical practice; and how CBCC fit well with their roles as community CHNs. Nurses’ responses to CBCC aligned closely with other elements of Rogers’ diffusion of preventative innovations theory, including relative advantage, complexity, trialability and observability [21]. Relative advantage is the degree to which an innovation is perceived to be advantageous or better than the idea it supersedes [21]. CHNs clearly saw CBCC as useful, valuable and worthwhile in their practice, as described in Theme 1. As CBCC provided the knowledge and tools to deliver better care, CHNs deemed it as advantageous, which likely contributed to its adoption in practice. Complexity describes the perceived level of difficulty to understand and use an innovation, with lower complexity associated with higher uptake [21]. This is reflected with CHNs describing CBCC as easy to understand, use and implement in practice, seemingly increasing its uptake. Trialability, the degree to which an innovation can be tested and adapted [21], was discussed by CHNs in terms of CBCC resources (when/how they were used) and key messages (when, to whom and how they were delivered). Hargreaves et al. describes this concept as ‘tinkering’; modifying an intervention or resource to suit the environment in which it is used [22]. CHNs described trialability in how they enlarged the ‘Dos and Don’ts’ poster from A4 to A3 size, so it would be easier for parents to read in child health clinic waiting rooms. They also expressed wanting more training on how to adapt delivery of CBCC messages depending on parents’ situation or needs. Finally, observability is the degree to which the effects of an innovation are visible to others [21]. Observability was important to nurses in this study, as they frequently discussed the perceived effects and benefits of CBCC in their practice and personal lives. For example, CHNs said the CBCC training increased their awareness of body image issues and their confidence in identifying and addressing such issues with parents; and noticed improvements in the quality and efficiency of their practice.

Our findings concur with previous research implementing healthy eating interventions with school nurses. A controlled trial by Pbert et al. (2013), conducted with seven nurses delivering a six-session counselling intervention to assist larger-bodied adolescents with changes in eating and physical activity, found 75% of nurses could incorporate the intervention into their daily work and found it acceptable for the adolescents they worked with [23]. Similarly, Al-Yateem and colleagues (2015) conducted a quasi-experimental trial of an intervention implemented by school nurses to increase adolescents’ knowledge of healthy eating [24]. Nurses presented a four-session education program to all adolescents aged 12–15 years in their school. Increases in student nutrition knowledge were observed in the intervention group compared to the control group after the intervention, however no examination of nurse experiences in implementing the program were explored [24]. Hence, while similar research exists suggesting health promotion interventions with nurses are feasible and useful, we believe our study is the first to implement an eating disorder prevention program in a community-based nurse setting to promote healthful eating and positive body image with parents of preschool children. Further, a 2018 systematic review of interventions for school nurses to improve student health outcomes [25] found of 65 included studies, only six (9%) were qualitative. Our study therefore also provides important insights to nurses’ experiences, which are rarely the focus of health implementation research.

### Future implications and recommendations

Early identification of body image, eating or weight issues among young children is integral to the early prevention of eating disorders, which cause significant burden to patients, families and health care organisations [26]. Hence, establishing health services with knowledgeable, skilled and confident CHNs who can discuss such issues and educate parents on evidence-based strategies to promote healthy attitudes towards body image and eating patterns is highly valuable. This may be seen as the role of dietitians and nutritionists; but with a lack of allied health staff across most Australian health services, particularly in community health settings, innovative models of care are essential to improve efficiency and effectiveness of health care. With educating and assessment of food, eating and growth being core business of community CHNs, they are ideally placed to expand their scope of practice to embed CBCC into their usual service delivery. Our study not only demonstrates that this can be done, but also shows that CHNs perceive CBCC to value-add to their clinical toolkit and improve care. More research is required to evaluate the use of CBCC in other community health settings and beyond, with innovations required to broaden its reach and impact, such as implementation in community health parent groups, kindergartens, schools or in mass media.

### Limitations

Despite useful findings, this study also has some important limitations. First, this is a small study of 11 CHNs at one health service in Australia, so it is possible that not all perceptions of CBCC were captured, and results may not be generalisable beyond the characteristics of our small sample. However, we did use maximum variation purposive sampling and continued data collection until saturation was reached, which may enable findings to be more broadly applicable. Second, the lead author (LN), who co-led the CBCC training workshop for CHNs, was previously a staff member in community child health at the health service, and conducted around half of the interviews. Given these multiple and close collaborations with the CHNs, interviewees may have been hesitant to express negative perceptions of CBCC. However, another author (SR), who was not known to participants, conducted the other half of interviews; and responses did not differ between interviewers, suggesting CHNs gave honest opinions. Because of the potential for desirability responding in this qualitative study, a quantitative analysis of nurse logbooks and CBCC resource use is also planned to triangulate the positive interview findings via objective measures.

## Conclusions

This study explored CHNs’ perceptions of the CBCC Program’s effectiveness (including an adapted training workshop, CBCC resources and key messages for open dialogue) in a community child health setting. CHNs were overwhelmingly accepting of CBCC, finding it useful and valuable in practice. Nurses reported an increased awareness of body image and noted efforts to change their language, both in their personal lives and in clinical practice. CHNs found some topics with some parents particularly difficult to broach and wanted ongoing education and training to address challenges and increase efficiency of CBCC delivery. More research is needed to broaden the reach and impact of CBCC, which appears to be a useful and effective tool for CHNs to deliver education to parents of young children for promoting positive body image and healthful eating patterns for the long-term prevention of eating disorders.

## Supplementary Information

**Additional file 1:** **Supplementary File 1**: Interview Guide.

## Data Availability

The datasets used and/or analysed during the current study are available from the corresponding author on reasonable request.

## Abbreviations

CBCC: Confident Body Confident Child
CCH: Community Child Health
CHN: Child Health Nurse
GCHHS: Gold Coast Hospital and Health Service
RCT: Randomised control trial
TDF: Theoretical Domains Framework
