# Supplementary material for: Child health nurse perceptions of using confident body, confident child in community health: a qualitative descriptive study
Source: BMC Nurs. 2020 Nov 6;19:103. doi: 10.1186/s12912-020-00499-7 (PMC7646073; doi:10.1186/s12912-020-00499-7)
Supplement: Supplementary file 1 — Additional file 1: Supplementary File 1: Interview Guide. [file 12912_2020_499_MOESM1_ESM.docx]

**Supplementary File 1: Interview Guide**

**Preamble:** “Thanks for taking the time to talk with me today. In this interview, I am going to ask you a few questions relating to your experiences with the CBCC program. We want to hear your story so we can understand what you about this program. There are no right or wrong answers. Before we start, do you have any questions about the interview?”

| **Semi-structured interview guide for Child Health Nurses** |
| --- |
| **Domain 1: CBCC resources** |
| 1. Did you use the CBCC resources/materials with parents? If so, which ones did you use the most and why? 2. Were there resources/materials you didn’t find particularly useful in clinic? If so, why? 3. Is there anything else you wish you had access to? 4. Do you intend to keep using CBCC resources with families? 5. Would you recommend CBCC resources/materials to other nurses? If so, which ones and why? |
| **Domain 2: Nurses training and clinical practice/skills** |
| 1. What do you remember most from the CBCC training? 2. What changes have you made to your clinical practice after attending the CBCC training? 3. Did your knowledge or confidence change in managing body image/healthy eating/weight issues with families? Which topics in CBCC helped you the most in your clinic? 4. Did the training workshop prepare you for using CBCC resources/materials in clinic? 5. How would you change the training to make it better for other nurses? 6. Was there anything that surprised you about the program or what you learned? 7. Were there any topics you would have liked more information about? 8. Did the program meet your expectations? |
| **Domain 3: Perceived value of the intervention for parents** |
| 1. What was your experience with using CBCC messages or resources with parents? [Prompts]:    1. What kind of feedback did you get from parents?    2. Did parents find them useful?    3. What parts were not useful to you? 2. Did you use the training or new knowledge in any other way with parents? |
| **Domain 4: Overall experience with intervention** |
| 1. What did you think about the CBCC program overall? 2. What did you like / what didn’t you like about the program? 3. Is there anything else you would like to comment on? |
| **Prompts:** If a question is answered with a simple yes or no, ask the patient to explain further. For example, you can ask “why” or “why not”?  Other generic prompts include: ““Can you tell me more about this?”; “Can you explain this further / expand on this?” and “What do you mean when you say ____?” |
